# USP18 Sensitivity of Peptide Transporters PEPT1 and PEPT2

**DOI:** 10.1371/journal.pone.0129365

**Published:** 2015-06-05

**Authors:** Jamshed Warsi, Zohreh Hosseinzadeh, Bernat Elvira, Lisann Pelzl, Ekaterina Shumilina, Dong-Er Zhang, Karl S. Lang, Philipp A. Lang, Florian Lang

**Affiliations:** 1 Department of Physiology I, University of Tuebingen, Tuebingen, Germany; 2 Department of Pathology & Division of Biological Sciences, Moores UCSD Cancer Center, University of California San Diego, La Jolla, CA, 92093, United States of America; 3 Institute of Immunology, Medical Faculty, University of Duisburg-Essen, Essen, Germany; 4 Department of Molecular Medicine II, University of Duesseldorf, Duesseldorf, Germany; University of Cambridge, UNITED KINGDOM

## Abstract

USP18 (Ubiquitin-like specific protease 18) is an enzyme cleaving ubiquitin from target proteins. USP18 plays a pivotal role in antiviral and antibacterial immune responses. On the other hand, ubiquitination participates in the regulation of several ion channels and transporters. USP18 sensitivity of transporters has, however, never been reported. The present study thus explored, whether USP18 modifies the activity of the peptide transporters PEPT1 and PEPT2, and whether the peptide transporters are sensitive to the ubiquitin ligase Nedd4-2. To this end, cRNA encoding PEPT1 or PEPT2 was injected into *Xenopus laevis* oocytes without or with additional injection of cRNA encoding USP18. Electrogenic peptide (glycine-glycine) transport was determined by dual electrode voltage clamp. As a result, in *Xenopus laevis* oocytes injected with cRNA encoding PEPT1 or PEPT2, but not in oocytes injected with water or with USP18 alone, application of the dipeptide gly-gly (2 mM) was followed by the appearance of an inward current (I_gly-gly_). Coexpression of USP18 significantly increased I_gly-gly_ in both PEPT1 and PEPT2 expressing oocytes. Kinetic analysis revealed that coexpression of USP18 increased maximal I_gly-gly_. Conversely, overexpression of the ubiquitin ligase Nedd4-2 decreased I_gly-gly_. Coexpression of USP30 similarly increased I_gly-gly_ in PEPT1 expressing oocytes. In conclusion, USP18 sensitive cellular functions include activity of the peptide transporters PEPT1 and PEPT2.

## Introduction

The Ubiquitin-like specific protease 18 (USP18), a de-ubiquitin enzyme [1, 2], interacts with interferon α (IFNα)- mediated signalling [3–13] and thus plays a decisive role in the anti-viral [1, 8, 14–16] and antibacterial [5, 17] immune response as well as autoimmune disease [18]. USP18 is mainly located in the cytosol whereas USP18-sf, an isoform of USP18 is distributed in both cytosol and nucleus [4]. USP18 is in part effective by modulating the transcription factors NF-κB and NFAT [19, 20]. Moreover, USP18 competitively inhibits IFN-α/β-induced JAK/STAT activation [13] and upregulates epidermal growth factor receptor (EGFR) expression [21]. USP18 counteracts apoptosis [11, 12, 22] and contributes to the signalling of tumorigenesis and anti-tumor immune response [3, 8, 23–25].

Ubiquitination plays a pivotal role in the regulation of several transport processes [26, 27]. Ubiquitination controls endocytosis and turnover of transport proteins [27]. An ubiquitin ligase particularly important for the regulation of channels and transporters is Nedd4-2 (neuronal precursor cell expressed developmentally downregulated 4–2), which down-regulates a wide variety of transport processes [26, 28–35]. At least in theory, those transporters could be targeted by de-ubiquitination enzymes. As a matter of fact, some transient receptor potential (TRP) channels are regulated by the de-ubiquitinating enzyme cylindromatosis (CYLD) [36]. Surprisingly, little is known about effects of de-ubiquitinating enzymes on other transport systems. Specifically, to the best of our knowledge, a role of altered transport across the cell membrane in the pleotropic effects of USP18 has never been shown.

The present study explored whether USP18 influences the activity of peptide transporters 1 (PEPT1) and 2 (PEPT2), which accomplish electrogenic cellular uptake of di- and tripeptides [37–39] including peptide-like drugs [37, 38]. Regulators of peptide transporters include glucocorticoids [40], leptin [41] and growth hormone [42].

In order to test for an effect of USP18 on peptide transporters, cRNA encoding PEPT1 and PEPT2 were injected into *Xenopus laevis* oocytes with or without additional injection of cRNA encoding USP18. Subsequently, peptide transport was derived from peptide induced current.

## Materials and Methods

### Ethics Statement

All animal experiments were conducted according to the recommendations of the Guide for Care and Use of Laboratory Animals of the National Institutes of Health as well as the German law for the welfare of animals, and reviewed and approved by the respective government authority of the state Baden-Württemberg (Regierungspräsidium) prior to the start of the study (Anzeige für Organentnahme nach §6). The Xenopus oocytes were explanted from adult Xenopus laevis (NASCO, Fort Atkinson, USA). The frogs were anaesthesized by a 0.1% Tricain solution. After confirmation of anaesthesia and disinfection of the skin, a small abdominal incision was made and oocytes were removed, followed by closure of the skin by sutures. All efforts were made to minimize animal suffering.

### Constructs

Constructs encoding rabbit PEPT1 or PEPT2 [43], human USP18 [14], human USP30 [44] and human KCNQ1/E1 or Kv7.1 [45] were used for generation of cRNA as described previously [43, 46].

### Voltage clamp

*Xenopus* oocytes were prepared as previously described [47]. Where not indicated otherwise, 10 ng cRNA encoding PEPT1, 20 ng cRNA encoding PEPT2, 12 ng cRNA encoding KCNQ1/E1 (9 ng KCNQ1 + 3 ng KCNE1), 10 ng cRNA encoding USP18 or 10 ng cRNA encoding USP30 were injected on the same day after preparation of the oocytes [48]. The oocytes were maintained at 17°C in ND96 solution containing (in mM): 88.5 NaCl, 2 KCl, 1 MgC1_2_, 1.8 CaC1_2_, 2.5 NaOH, 5 HEPES (pH 7.4), sodium pyruvate (C_3_H_3_NaO_3_), Gentamycin (100 mg/l), Tetracycline (50 mg/l), Ciprofloxacin (1.6 mg/l), and Theophiline (90 mg/l). The voltage clamp experiments were performed at room temperature 3 days after injection of cRNA encoding PEPT1 and USP18 or 4 days after injection of cRNA encoding PEPT2 and USP18. Two-electrode voltage-clamp recordings were performed at a holding potential of -70 mV. The data were filtered at 10 Hz and recorded with a Digidata A/D-D/A converter (1322 Axon Instruments) and Clampex 9.2 software for data acquisition and analysis (Axon Instruments) [49]. The control superfusate (ND96) contained (in mM): 93.5 NaCl, 2 KCl, 1.8 CaCl_2_, 1 MgCl_2_, 2.5 NaOH and 5 HEPES, pH 7.4. [50]. Glycine-glycine was added to the solutions at a concentration of 2 mM, unless otherwise stated [51]. The flow rate of the superfusion was approx. 20 ml/min, and a complete exchange of the bath solution was reached within about 10 s [52]. The peptide induced current was in preliminary experiments 2.5 ± 0.9 nA (n = 9) 3 days and 13.1 ± 1.5 nA (n = 16) 4 days after injection of 20 ng cRNA encoding PEPT2. The peptide induced current was in preliminary experiments 3.9 ± 1.3 nA (n = 8) and 12.3 ± 1.9 nA (n = 16) 4 days after injection of 10 ng or 20 ng, respectively, cRNA encoding PEPT2.

### Detection of PEPT2-HA cell surface expression by chemiluminescence

To determine PEPT2-HA cell surface expression by chemiluminescence [53], defolliculated oocytes were first injected with 20 ng cRNA encoding either PEPT2-HA and/or 10 ng cRNA encoding USP18. After 4 days of incubation, oocytes were blocked with 1% BSA in ND96 solution for 20 minutes and then incubated with anti-HA-HRP antibody (diluted 1:1000, Miltenyi Biotec, Germany). Next, oocytes were washed three times 10 minutes each with 1% BSA in ND96 solution followed by three times 10 minutes each in ND96 solution. Individual oocytes were placed in 96 well plates with 20 μl of SuperSignal ELISA Femto Maximum Sensitivity Substrate (Pierce, Rockford, IL, USA), and chemiluminescence of single occytes was quantified in a luminometer (Walter Wallac 2 plate reader, Perkin Elmer, Juegesheim, Germany) by integrating the signal over a period of 1 s. Results display normalized relative light units.

### Statistical analysis

Data are provided as means ± SEM, n represents the number of oocytes investigated. All voltage clamp experiments were repeated with at least 3 batches of oocytes; in all repetitions qualitatively similar data were obtained. Data were tested for significance using ANOVA (Tukey test or Kruskal-Wallis test) or t-test, as appropriate. Results with p < 0.05 were considered statistically significant.

## Results

In order to test for an effect of USP18 on the activity of the peptide transporters, the peptide transporters PEPT1 or PEPT2 were expressed in *Xenopus laevis* oocytes with or without additional expression of USP18. The inward current observed following addition of the dipeptide glycine-glycine (2 mM) to the bath solution (I_gly-gly_) was taken as a measure of peptide transport.

**Fig 1:**
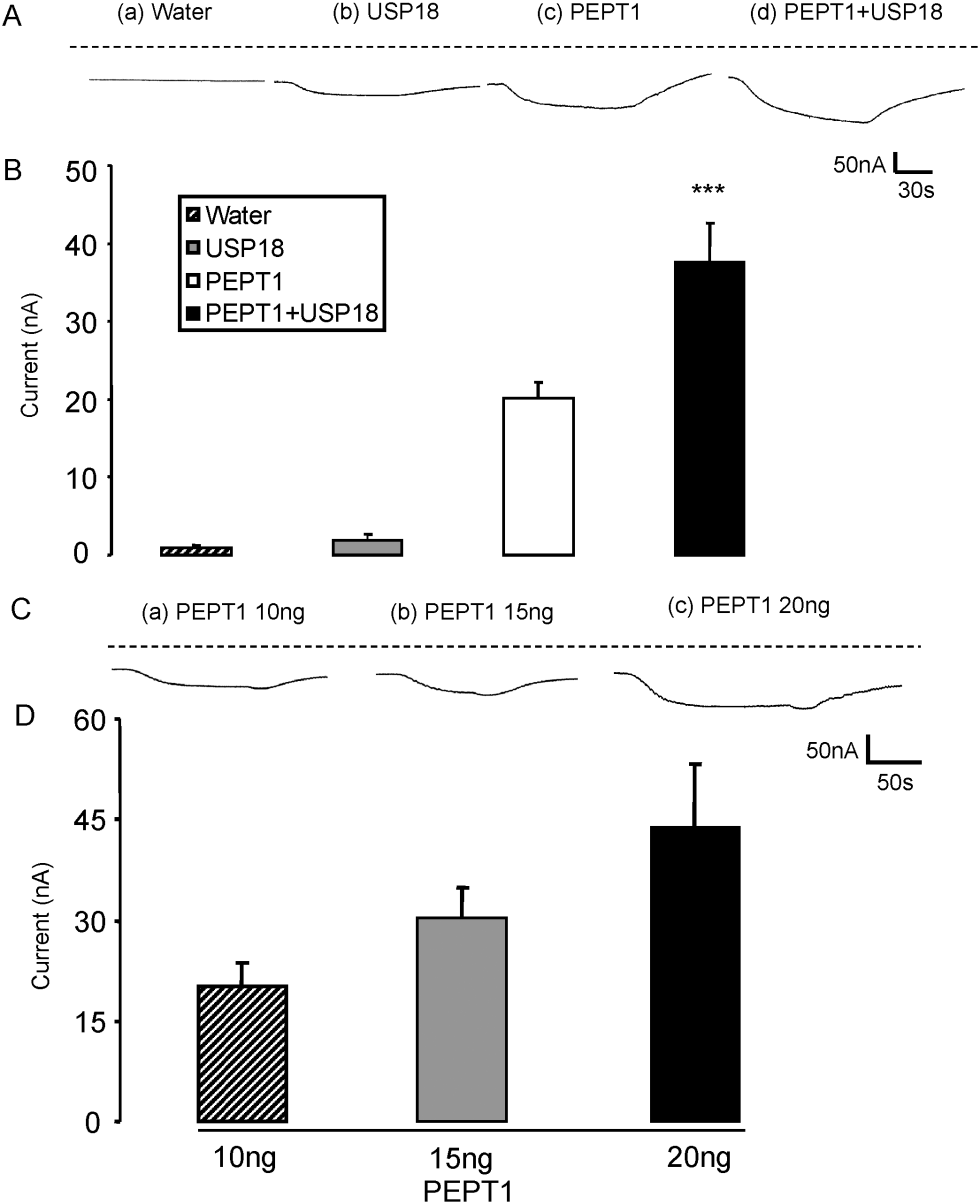
Coexpression of USP18 increases electrogenic peptide transport in PEPT1-expressing *Xenopus laevis* oocytes. **A:** Representative original tracings showing I_gly-gly_ in *Xenopus laevis* oocytes injected with water (a), expressing USP18 alone (b) or expressing PEPT1 without (c) or with additional coexpression of wild type USP18 (d). **B:** Arithmetic means ± SEM (n = 12–18) of I_gly-gly_ in *Xenopus laevis* oocytes injected with water (striped bar), or expressing USP18 alone (grey bar) or expressing PEPT1 without (white bar) or with (black bar) USP18. **C:** Representative original tracings showing glycine-glycine (2 mM)—induced current (I_gly-gly_) in *Xenopus laevis* oocytes injected with (a) 10 ng, (b) 15ng, or (c) 20ng cRNA encoding PEPT1. **D:** Arithmetic means ± SEM (n = 9–10) of I_gly-gly_ in *Xenopus laevis* oocytes injected with 10 ng (striped bar) or 15 ng (grey bar) or 20 ng (black bar) cRNA encoding PEPT1. *** (p<0.001) indicates statistically significant difference from the absence of USP18.

I_gly-gly_ was negligible in water-injected *Xenopus laevis* oocytes (Fig 1A and 1B). Similarly, injection of cRNA encoding USP18 alone (Fig 1A and 1B) did not yield appreciable I_gly-gly_. Accordingly, neither in the presence nor in the absence of USP18, *Xenopus laevis* oocytes expressed sizable endogenous electrogenic glycine-glycine transport. In contrast, addition of glycine-glycine to the bath resulted in the appearance of I_gly-gly_ in *Xenopus laevis* oocytes injected with cRNA encoding PEPT1. I_gly-gly_ increased with increasing amounts of cRNA injected (Fig 1C and 1D). The additional injection of cRNA encoding wild type USP18 significantly increased I_gly-gly_ in PEPT1 expressing *Xenopus* oocytes (Fig 1A and 1B).

**Fig 2:**
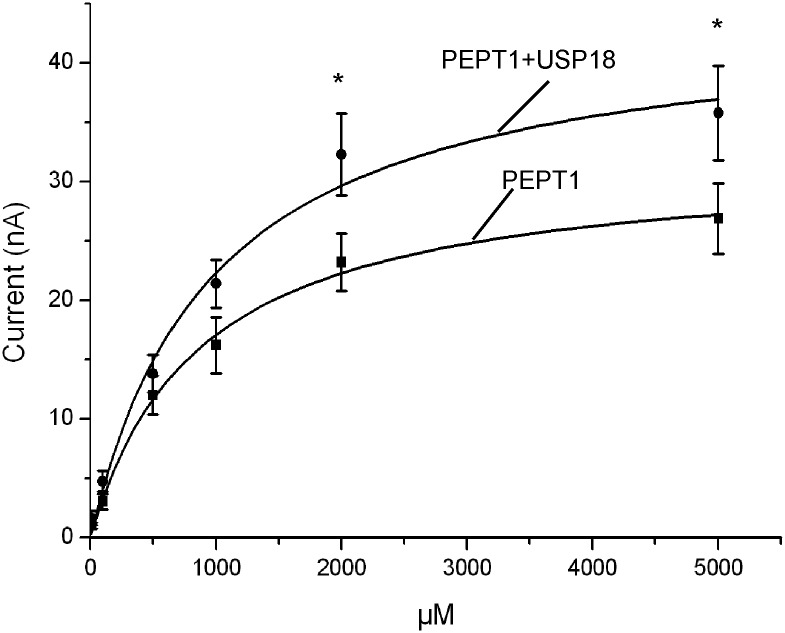
Coexpression of USP18 increases maximal electrogenic peptide transport in PEPT1-expressing *Xenopus laevis* oocytes. Arithmetic means ± SEM (n = 11–12) of I_gly-gly_ as a function of glycine-glycine concentration in *Xenopus laevis* oocytes expressing PEPT1 without (black squares), or with (black circles) additional coexpression of wild type USP18. *(p<0.05) indicates statistically significant difference from the absence of USP18.

In order to test whether USP18 was effective by modifying maximal transport rate and/or affinity of PEPT1, the oocytes were exposed to glycine-glycine concentrations ranging from 10 μM to 5 mM. As shown in Fig 2 an increase of the bath peptide concentration was followed by an increase of I_gly-gly_ in both, *Xenopus* oocytes expressing PEPT1 alone and *Xenopus* oocytes expressing PEPT1 and USP18. The increase of I_gly-gly_ was, however, larger in *Xenopus laevis* oocytes expressing PEPT1 with USP18 than in *Xenopus laevis* oocytes expressing PEPT1 alone. Kinetic analysis yielded apparent maximal currents, which were significantly (p<0.05) higher in *Xenopus laevis* oocytes expressing both, PEPT1 and USP18 (47.7 ± 5.5 nA, n = 11–12) than in *Xenopus laevis* oocytes expressing PEPT1 alone (32.5 ± 3.3 nA, n = 11–12). The glycine-glycine concentrations required for half maximal current (K_**M**_) were not significantly different between *Xenopus laevis* oocytes expressing PEPT1 alone (1297 ± 379 μM, n = 11–12) and in *Xenopus laevis* oocytes expressing PEPT1 together with USP18 (1038 ± 160 μM, n = 11–12). Thus, USP18 did not significantly modify the affinity of PEPT1.

**Fig 3:**
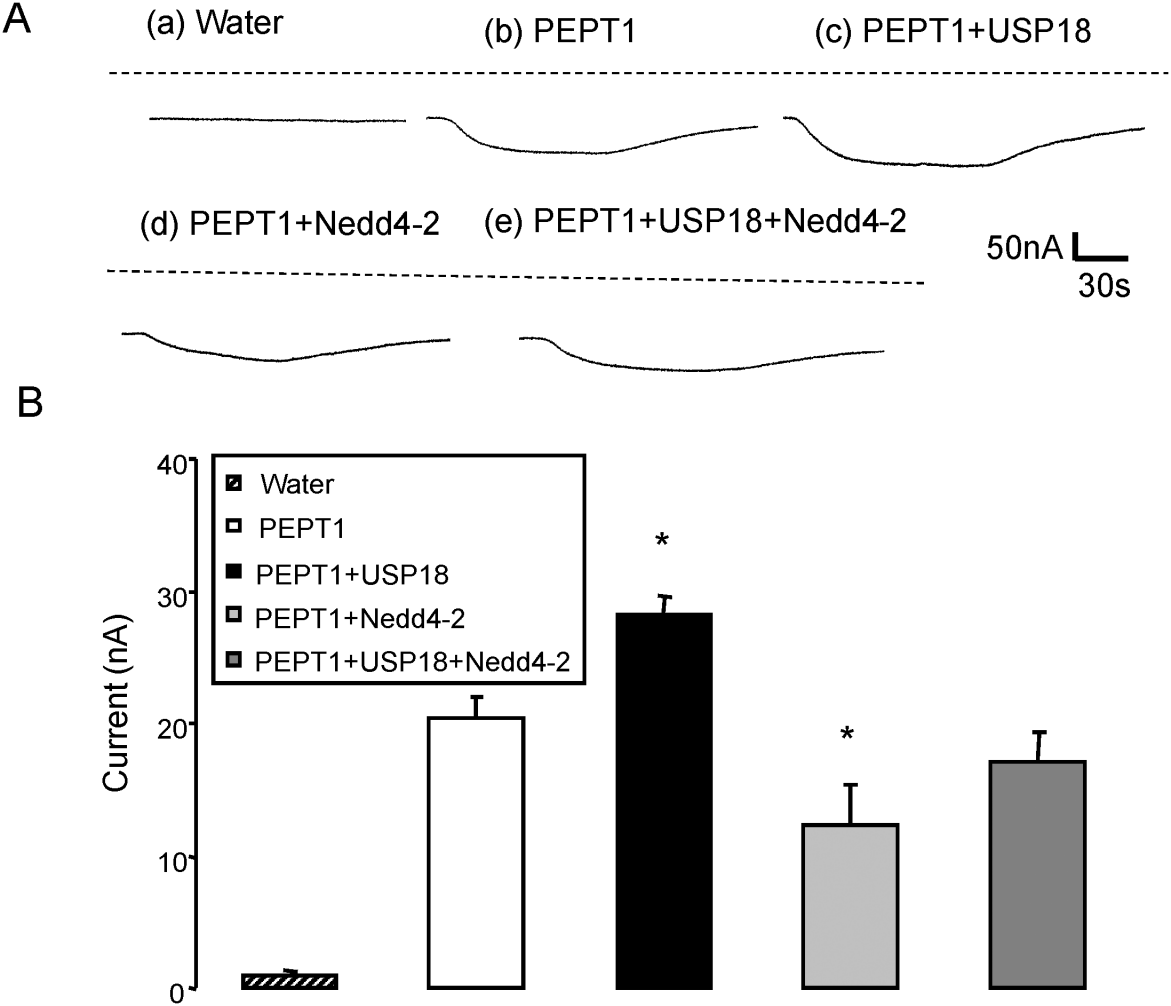
Coexpression of Nedd4-2 decreases electrogenic peptide transport in PEPT1-expressing *Xenopus laevis* oocytes. **A:** Representative original tracings showing I_gly-gly_ in *Xenopus laevis* oocytes injected with water (a), expressing PEPT1 alone (b) or with USP18 (c), Nedd4-2 (d), or with USP18 and Nedd4-2 (e). **B:** Arithmetic means ± SEM (n = 13–15) of I_gly-gly_ in *Xenopus laevis* oocytes injected with water (striped bar) or expressing PEPT1 without (white bar) or with USP18 (black bar), with Nedd4-2 (light grey bar), or with USP18 and Nedd4-2 (dark grey bar). *(p<0.05) indicates statistically significant difference from oocytes expressing PEPT1 alone.

Additional experiments addressed the sensitivity of I_gly-gly_ to the ubiquitin ligase Nedd4-2. To this end, PEPT1 or PEPT1 + USP18 were expressed without and with additional expression of Nedd4-2. As illustrated in Fig 3, coexpression of Nedd4-2 significantly (p<0.05) decreased I_gly-gly_ in both, PEPT1 and USP18 expressing *Xenopus laevis* oocytes and in *Xenopus laevis* oocytes expressing PEPT1 without USP18.

**Fig 4:**
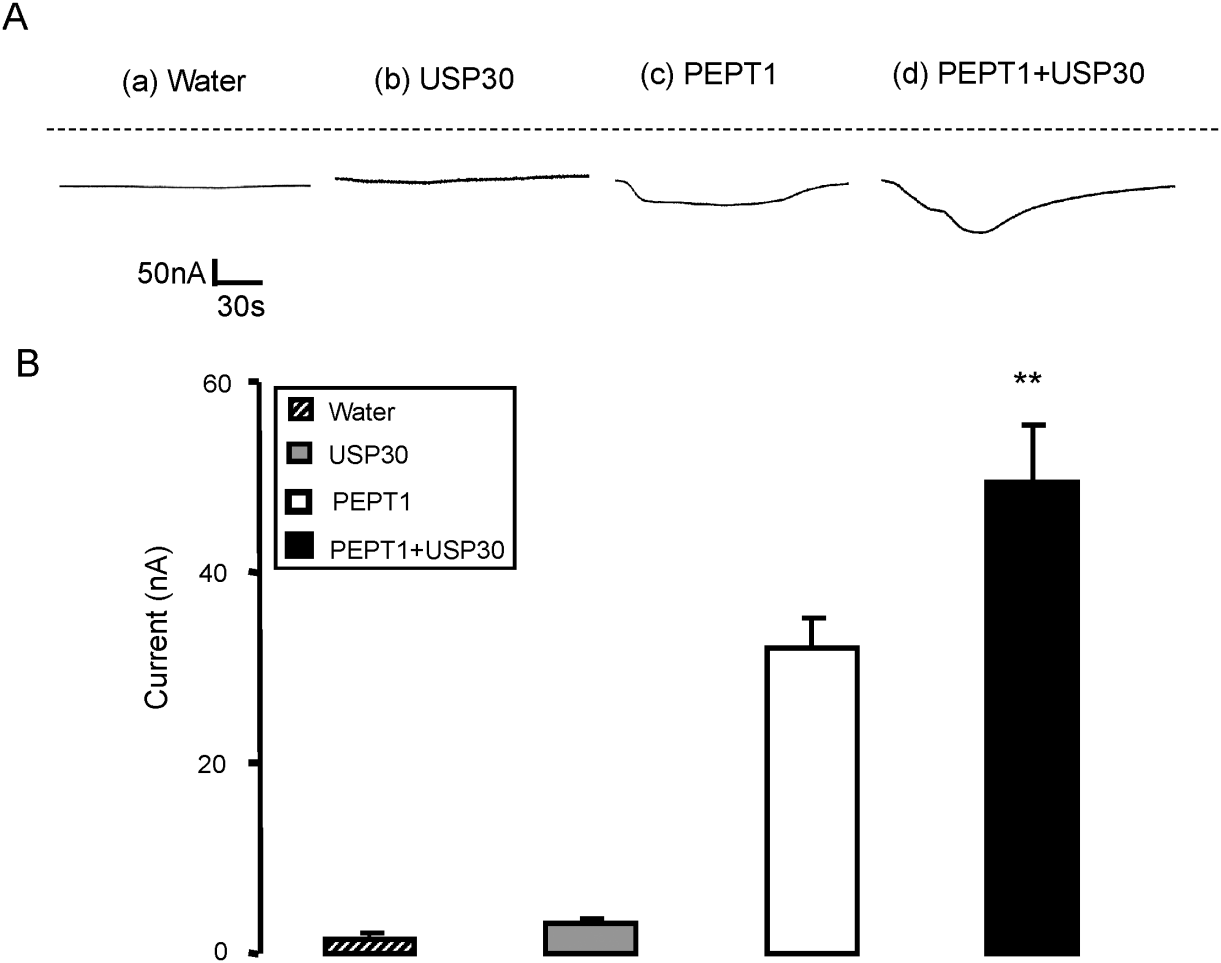
Coexpression of USP30 increases electrogenic peptide transport in PEPT1-expressing *Xenopus laevis* oocytes. **A:** Representative original tracings showing I_gly-gly_ in *Xenopus laevis* oocytes injected with water (a), expressing USP30 alone (b) or expressing PEPT1 without (c) or with additional coexpression of wild type USP30 (d). **B:** Arithmetic means ± SEM (n = 14–16) of I_gly-gly_ in *Xenopus laevis* oocytes injected with water (striped bar), or expressing USP30 alone (grey bar) or expressing PEPT1 without (white bar) or with (black bar) USP30. ** (p<0.01) indicates statistically significant difference from the absence of USP30.

A separate series of experiments explored the effect of USP30, another member of deubiquitinating protease family of enzymes, on the peptide transporter PEPT1. In *Xenopus* oocytes expressing PEPT1, the addition of glycine-glycine to the bath was again followed by the appearance of I_gly-gly_, which was significantly (p<0.01) enhanced by additional injection of cRNA encoding USP30 (Fig 4).

**Fig 5:**
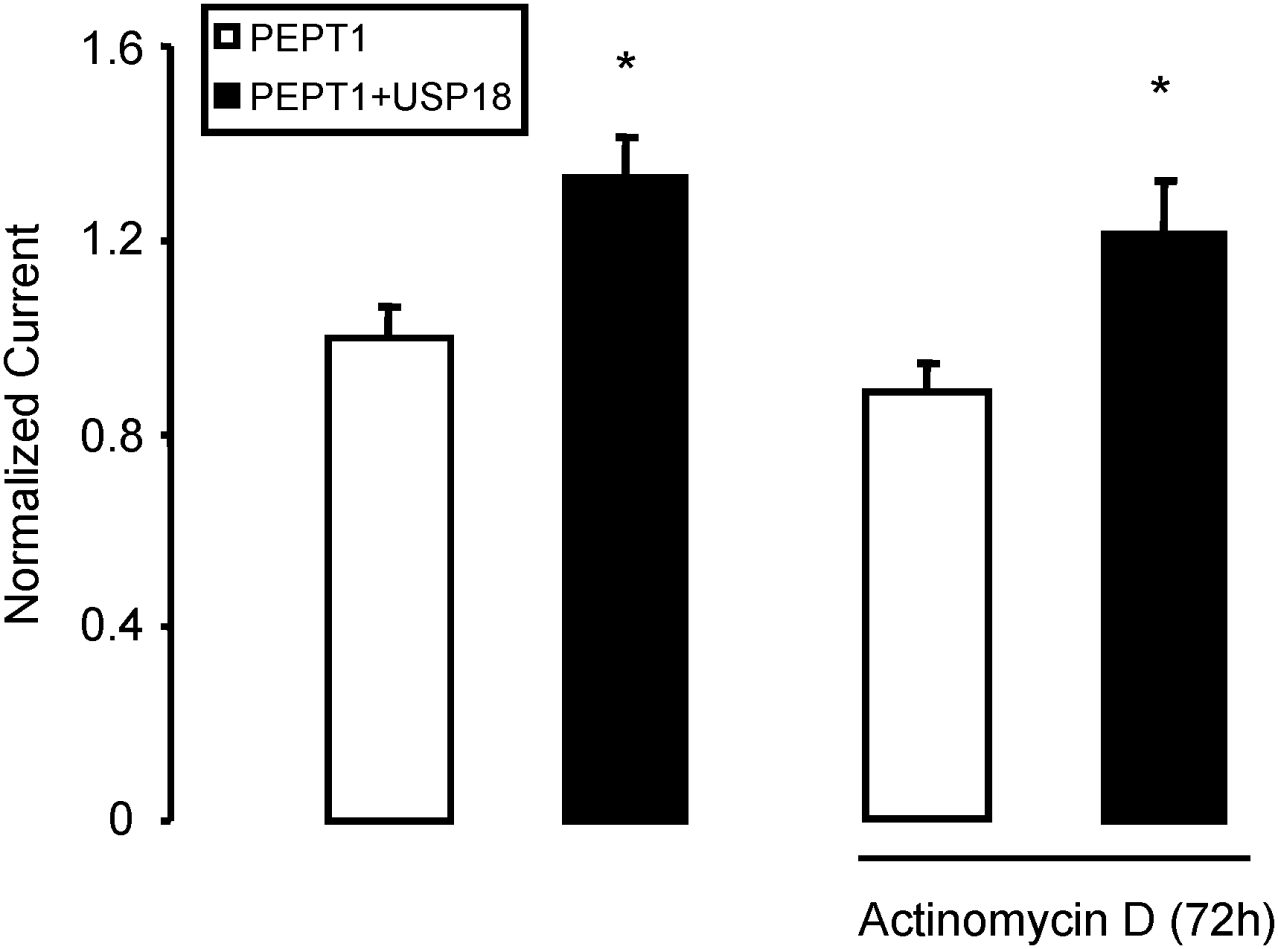
Effect of USP18 on PEPT1 in absence and presence of Actinomycin D. Arithmetic means ± SEM (n = 15–16) of I_gly-gly_ in *Xenopus* oocytes injected PEPT1 without (white bar) or with (black bar) wild type USP18 in the absence (left bars) and presence (right bars) of 50 nM Actinomycin D 72 hours prior to measurement. *(p<0.05) indicates statistically significant difference from the absence of USP18.

In order to test whether the effect of USP18 on I_gly-gly_ required transcription, experiments were performed in the presence of actinomycin D (50 nM, added 72 hours prior to the experiment). Inhibition of transcription by incubation (72 hours) with actinomycin D (50 nM) did not significantly modify I_gly-gly_ in presence or absence of USP18 (Fig 5).

**Fig 6:**
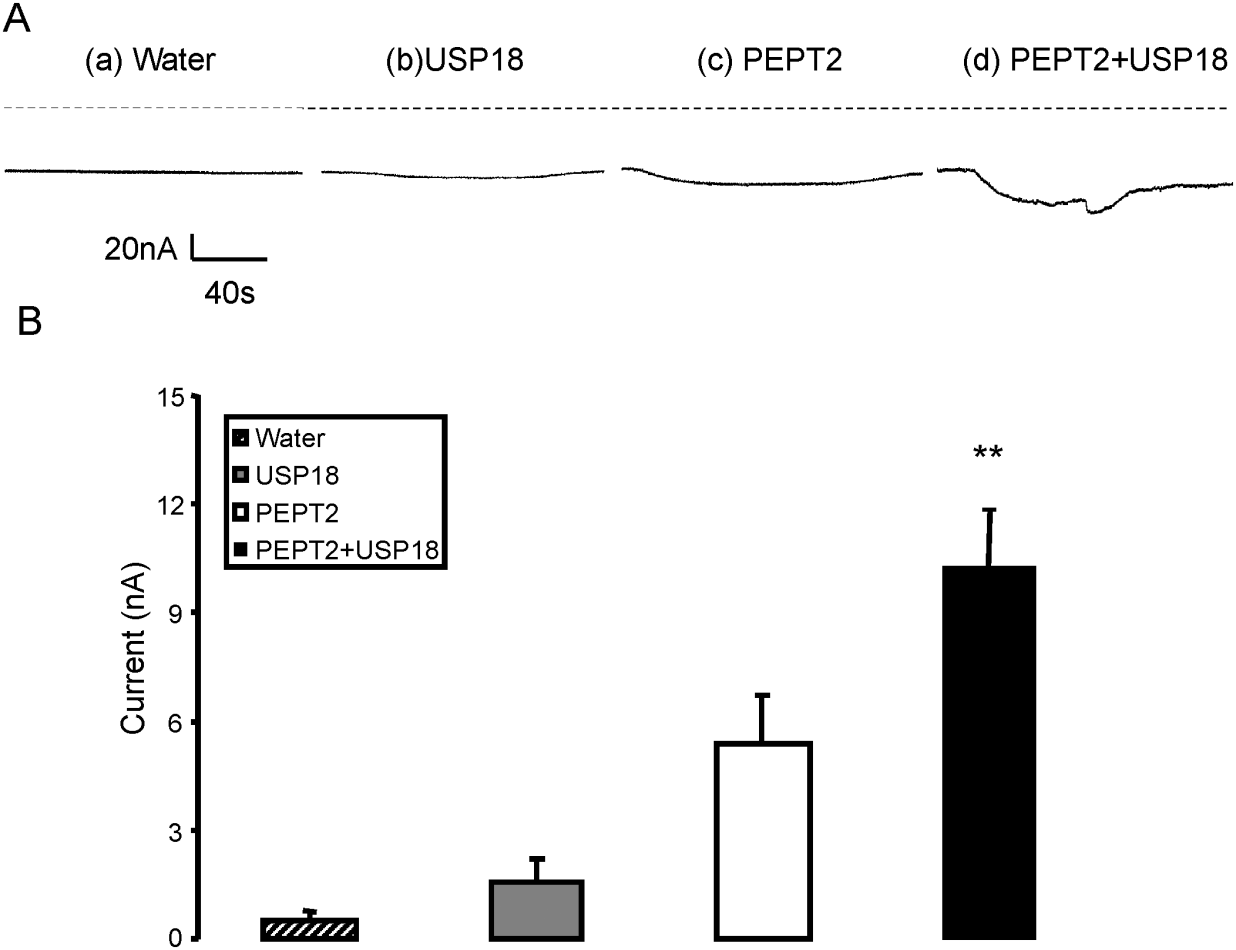
Coexpression of USP18 increases electrogenic peptide transport in PEPT2-expressing *Xenopus laevis* oocytes. **A:** Representative original tracings showing I_gly-gly_ in *Xenopus laevis* oocytes injected with water (a) or expressing USP18 alone (b) or expressing PEPT2 without (c) or with additional coexpression of wild type USP18 (d). **B:** Arithmetic means ± SEM (n = 8–9) of I_gly-gly_ in *Xenopus* oocytes injected with water (striped bar), expressing USP18 alone (grey bar), or expressing PEPT2 without (white bar) or with (black bar) wild type USP18. **(p<0.01) indicates statistically significant difference from the absence of USP18.

A further series of experiments explored the putative effects of USP18 on the peptide transporter isoform PEPT2. Similar to what has been observed in *Xenopus* oocytes expressing PEPT1, in *Xenopus* oocytes expressing PEPT2 addition of glycine-glycine to the bath was followed by the appearance of I_gly-gly_ (5.4 ± 1.3 nA), which was significantly (p<0.05) enhanced by additional injection of cRNA encoding USP18 (10.2 ± 1.6 nA) (Fig 6).

Chemiluminescence was employed to quantify PEPT2 protein abundance in the cell membrane. The protein abundance in the oocytes was, however, similar in oocytes co-expressing PEPT2-HA with USP18 (1.04 ± 0.05 relative light units, n = 113) and in oocytes expressing PEPT2-HA alone (1.00 ± 0.03 relative light units, n = 116).

A further series of experiments explored, whether the voltage gated K^**+**^ channel KCNQ1/E1 was sensitive to USP18. As a result, the current at +80mV was negligible in *Xenopus laevis* oocytes injected with water (1.2 ± 2.2 nA, n = 139) and was similarly high in *Xenopus laevis* oocytes injected with KCNQ1/E1 alone (190.3 ± 17.5 nA, n = 19) and in *Xenopus laevis* oocytes injected KCNQ1/E1 and USP18 (180.1 ± 10.7 nA, n = 19).

## Discussion

The present study reveals a completely novel function of the de-ubiquitin enzyme USP18, i.e. the up-regulation of the peptide transporter isoforms, PEPT1 and PEPT2. The effect was mimicked by USP30 and may thus be a hitherto unknown function of several USP isoforms.

It is tempting to speculate that USP18 is effective by reversing the ubiquitination and subsequent degradation of the carrier protein. Accordingly, USP18 apparently enhances the maximal transport rate. A role of ubiquitination in the regulation of peptide transporters is further suggested by the experiments with Nedd4-2. Coexpression of the ubiquitin ligase Nedd4-2 tended to down-regulate the peptide transporter PEPT1. Comparison of the currents in *Xenopus laevis* oocytes expressing PEPT1 and USP18 with *Xenopus laevis* oocytes expressing PEPT1, USP18 and Nedd4-2 suggest that the effect of USP18 might be overridden by the effect of Nedd4-2. It must be kept in mind, though, that the effect of USP18 may be unrelated to that of Nedd4-2.

USP18 did not appreciably modify the protein PEPT2 protein abundance in the cell membrane. It cannot be excluded that USP18 is effective by mechanisms other than the de-ubiquination of the carrier protein. The effect of USP18 on peptide transport apparently does not require transcription. In theory, USP18 could modify peptide transporters by modifying the activity of proteins regulating peptide transporters. Signalling involved in the regulation of peptide transporters include phosphoinositide (PI) 3 kinase [54], phosphoinositide dependent kinase PDK1 [54], serum & glucocorticoid inducible kinase SGK1 [55] and AMP activated kinase [56].

The peptide transporters PEPT1 and PEPT2 accomplish the cellular uptake of di- and tripeptides [37–39] and several drugs [37, 56–58] including beta-lactam antibiotics, angiotensin-converting enzyme inhibitors, antiviral drugs, and anti-cancer agents [38, 59–63]. The carriers are expressed in a variety of cells including proximal renal tubules [64], enterocytes [38, 61], cancer cells [65, 66], and immune cells, such as macrophages [67–69]. The *in vivo* relevance of USP18 in the regulation of peptide transport remains, however, to be shown. It would be particularly interesting to learn, whether or not USP18 sensitivity of cellular peptide transport is relevant for antiviral and antibacterial activity of USP18.

In conclusion, USP18 has the potency to enhance the activity of the peptide transporters PEPT1 and PEPT2. Further experiments are needed to define the *in vivo* significance of USP18 sensitive peptide transport.
